# Revolutionizing faculty’s intrinsic work motivation in China: A novel serial mediation model integrating value-based leadership, growth mindset, and teaching self-efficacy

**DOI:** 10.1371/journal.pone.0313392

**Published:** 2025-01-14

**Authors:** Xiangge Zhao, Pei Yang, Xinxin Zhang, Nanzhe Li

**Affiliations:** School of Foreign Languages for International Business, Hebei Finance University, Baoding, Hebei, China; University of Central Punjab, PAKISTAN

## Abstract

This study presents a revolutionary understanding of how value-based leadership enhances the intrinsic work motivation of Chinese university faculty. A novel serial mediation model is introduced, highlighting the interplay between growth mindset and teaching self-efficacy in transmitting the impact of leadership to increased intrinsic motivation. Utilizing a comprehensive sample of 394 faculty members from across China, advanced SmartPLS 3.0 analytics were employed to validate the model. The results demonstrate a significant positive effect of value-based leadership on faculty intrinsic motivation, partially mediated by the sequential influence of nurturing a growth mindset and strong teaching self-efficacy. The findings provide fresh theoretical perspectives and practical strategies for researchers, university leaders, administrators, and policymakers seeking to elevate the intrinsic work motivation of China’s academic faculty to new levels.

## Introduction

Work motivation has garnered increased scholarly attention within the educational context since the turn of the millennium, with a notable surge in research across diverse sociocultural backgrounds over the past decade [1–3]. China’s higher education system, instrumental in fostering skilled professionals and experts crucial to societal progress, contributes significantly to the nation’s economic growth, technological advancements, scientific achievements, and societal evolution [4, 5]. Despite these accomplishments, research has highlighted that faculty members (or “faculty” for short in some countries, sharing the same meaning with “faculty members” in this study) in China often lack sufficient work motivation and are not fully dedicated to their teaching responsibilities [6, 7]. Understanding and enhancing faculty work motivation is imperative for fostering teaching engagement, academic research, and sustainable university development [1, 8].

Work motivation can be broadly classified into extrinsic and intrinsic forms. While extrinsic motivation is tied to external rewards and punishments, intrinsic motivation stems from personal interest or enjoyment in the task itself [9]. A growing body of evidence suggests that intrinsic motivation plays a pivotal role in enhancing employee performance and well-being, particularly among faculty, whose profession thrives on intrinsic drives [9–11]. Intrinsically motivated faculty members tend to invest more time and effort into teaching, demonstrating innovative classroom activities, actively seeking feedback, and embracing cutting-edge educational techniques [12, 13]. However, the effectiveness of strategies to promote intrinsic motivation varies contextually and culturally [1, 14].

Given China’s unique sociocultural backdrop steeped in Confucianism, which emphasizes collectivism, interpersonal harmony, deference to authority, and virtue, it is imperative to explore factors that influence faculty’s intrinsic work motivation within this cultural context [15]. This research proposes value-based leadership as a key strategy to elevate faculty’s intrinsic motivation, grounded in the notion that values are fundamental drivers of motivation [16]. Values are deeply ingrained in the teaching profession, and in China, where Confucian educational philosophies persist [17], leaders’ values are perceived as paramount [18]. Thus, value-based leadership may effectively bolster faculty’s intrinsic work motivation in this cultural setting.

Moreover, this study delves into the sequential mediating effects of faculty’s growth mindset and teaching self-efficacy on the relationship between value-based leadership and intrinsic work motivation. A growth mindset fosters adaptability, progress, and an open-minded approach to learning [19]. Teaching self-efficacy, on the other hand, represents faculty’s confidence in their teaching abilities [20, 21]. These two factors potentially serve as psychological bridges linking university organizational dynamics with faculty’s intrinsic work motivation [22]. However, limited research has examined how value-based leadership interacts with personal attributes like growth mindset and teaching self-efficacy to shape faculty’s intrinsic work motivation in Chinese universities [23]. Therefore, this study aims to bridge this gap by investigating the impact of value-based leadership on faculty’s intrinsic work motivation, with a focus on the serial mediation of growth mindset and teaching self-efficacy.

Guided by these considerations, the research questions formulated are:

Q1: To what degree does value-based leadership influence faculty’s intrinsic work motivation in Chinese universities?
Q2: Is the relationship of value-based leadership and faculty’s intrinsic work motivation sequentially mediated by their growth mindset and teaching self-efficacy?

## Literature review

### Intrinsic Work Motivation (IWM)

Faculty’s intrinsic motivation encompasses various factors that propel educators to embark on and persist in a teaching career, enabling them to thrive within it [1]. Drawing upon Self-determination Theory (SDT) [24], individuals inherently strive for psychological growth, integration, learning, mastery, and connection with others. Hence, three fundamental psychological needs—autonomy, competence, and relatedness—are recognized as key drivers of self-motivated behavior [24]. Autonomy refers to the desire to act as an independent agent in one’s life [25], nourished by experiences that evoke interest and value, and diminished by external control, irrespective of incentives or punishments [9]. Competence encompasses the capability to manage outcomes and attain mastery in an activity [25], with ideal environments fostering this need through appropriate challenges, positive feedback, and opportunities for growth [26]. Relatedness pertains to the longing for interaction, belonging, and connection, nurtured by expressions of respect and empathy [9].

Previous research has delved into the influence of various variables, including social context factors, on intrinsic work motivation. Core social context elements involve organizational support and hindrances that impact employees’ fundamental psychological needs for competence, relatedness, and autonomy, with these being significantly shaped by management styles [27]. Many studies centering on workplace factors that may influence motivational variables have zeroed in on managerial actions, particularly leadership [18, 27, 28]. Established theories such as SDT [26], Goal-setting Theory [29], Expectancy Theory [30], and the Job Characteristics Model (e.g. [31, 32]) offer explanations on how leadership affects work motivation [33].

### Value-based leadership and intrinsic work motivation

Over the past two decades, educational leadership has garnered increased attention [34, 35]. There is a substantial and growing body of empirical research on educational leadership and management, particularly well-represented in regions such as Latin America, Asia, and Africa [36]. Recently, it has been proposed that incorporating indigenous cultural perspectives into educational leadership studies could enhance their value, as the “hidden effects” of socio-cultural contexts significantly influence motivating outcomes [4, 28]. While there are numerous studies examining Chinese leadership through the lens of Confucian virtues in non-educational settings (e.g. [37, 38]), research exploring the connection between Chinese value-based leadership and Confucian culture within higher education institutions in China remains limited [28].

[39] describe value-based leadership (VBL) as an engagement that sets goals, solves problems, creates language, and develops values, and is grounded in the organization’s values and high ethical standards. There is general agreement that VBL emphasizes shared values and is based on ethical and moral principles [40–42]. Confucianism lays a foundation for collectivism in Chinese culture, encouraging individuals to prioritize group benefits when there is a conflict between group and individual benefits [43, 44]. Faculty are consequently expected to strive for the realization of university’s goals with individual benefits sometimes being sacrificed [45]. In addition, Chinese culture is on high power distance and hierarchy is respected in management [43], resulting in a top-down policy-making model, in which senior managers usually establish policies by themselves and faculty members play a minor role in the process of policy-making [45, 46]. University leaders may utilize VBL to drive visible outcomes via invisible values since values are the unseen force that affect how people act, make decisions, interact with others, and choose their behaviors [41, 47]. Therefore, VBL is potentially an appropriate leadership style in Chinese socio-cultural context that can align organizational values, leaders’ values and followers’ values, thus motivate faculty members to fulfill organizational mission in a harmonious working atmosphere.

### Growth Mindset (GM)

An individual’s mindset encapsulates their overall approach to problem-solving and general attitude [48]. [19] distinguishes between two types of mindsets: growth mindset and fixed mindset. Growth mindset posits that all human attributes, including intelligence, can be nurtured and enhanced through dedication, hard work, and an effective strategy [19]. In contrast, individuals with a fixed mindset view intelligence as an inherent ability, fixed at birth. In contrast, those with a growth mindset are more hopeful about refining their skills or talents through greater effort, perceiving obstacles as inherent aspects of learning and striving for personal progress by persevering through challenging tasks [49, 50].

People endowed with a growth mindset are seldom satisfied with the status quo and constantly strive for improvement, driven not by external rewards but by an intrinsic motivation. This mindset fosters a desire to achieve one’s potential, encourages openness to learning, promotes staying abreast of emerging trends, and sustains a relentless quest for enhancement, fueling a continuous upward spiral of personal development [50, 51]. Value-based leadership is concerned with conveying the leader’s value system to the full group of employees with whom he works to consolidate the employees’ beliefs, assumptions, aspirations, and values, so establishing a supportive, respectful, and challenging environment that promotes the improvement of a growth mindset [52]. Leaders that practice value-based leadership are convinced that everyone can advance their knowledge and abilities while achieving greater success, and employees are thus urged to improve personally and professionally by going outside their comfort zones [53].

The environment of education which emphasizes continuous learning requires more awareness of the importance of growth mindset. Growth mindset was related to motivation in prior research, but mainly focusing on the relationship between growth mindset and students’ academic motivation, or how students’ mindset, enthusiasm for learning, and academic success are affected by teachers’ mindset [54–56]. There is little research on how faculty’s mindset impacts their intrinsic work motivation [57], also limited empirical research on the effect of value-based leadership on faculty’s growth mindset.

### Teaching Self-Efficacy (TSE)

Efficacy encompasses the capability and effectiveness in achieving desired outcomes, while self-efficacy pertains to an individual’s perception of their own abilities and effectiveness in a specific context [58]. Self-efficacy significantly influences how individuals confront challenges and the level of effort they exert [59]. Specifically, teaching self-efficacy among faculty members signifies their confidence in their proficiency to succeed in teaching-related endeavors [20, 21].

[60] highlights four key sources that shape an individual’s self-efficacy: vicarious experiences, mastery experiences, social persuasions, and personal physiological or emotional states. Vicarious experiences mean people make inferences about themselves through observing others’ actions, which are viewed as a model to predict their own success or failure in similar activities [60]. Others with similar capabilities succeeding in similar tasks will increase observers’ self-efficacy and convince them that they are also probably to be successful like others [60]. Leaders are usually successful persons in their domains, so they set up a role model for their subordinates, who will predict their own success in similar activities, thus their self-efficacy will be enhanced [61]. According to the Job Demands-Resources (JD-R) Theory [62], self-efficacy is one of the most important work resources. It can get individuals more motivated in their work, as individuals with better self-efficacy are more positive with less stress, anxiety and depression [60].

[53] justified the positive effect of teachers’ servant leadership on learners’ growth mindset, revealing the relations between leadership and growth mindset. [63] proved that both transformational leadership and growth mindset had positive impact on teacher self-efficacy by examining a sample of 1297 teachers in China. While previous research has offered valuable insights into the interplay of leadership styles, growth mindset, and self-efficacy, a deeper exploration is necessitated to understand how organizational factors, specifically value-based leadership, interact with personal factors such as growth mindset and teaching self-efficacy to shape faculty’s intrinsic work motivation. This exploration is particularly crucial within the distinct Chinese cultural context, which is heavily influenced by Confucian values.

Value-based leadership, when aligned with Confucian virtues, can cultivate an environment that nurtures faculty’s intrinsic motivation by instilling a sense of contributing to a larger, meaningful purpose. Furthermore, the Confucian emphasis on lifelong learning inherently fosters a growth mindset, motivating educators to persevere and learn from challenges. This mindset subsequently bolsters teaching self-efficacy, as faculty gain confidence in their capacity to positively influence student learning. As a result, their intrinsic motivation is further amplified by the fulfillment derived from witnessing their students’ progress.

This study seeks to delve into the intricate interplay between intrinsic work motivation, value-based leadership, growth mindset, and teaching self-efficacy, all within the framework of China’s Confucian culture. By doing so, it aims to provide a more comprehensive understanding of the mechanisms that drive faculty’s intrinsic motivation in this unique cultural context.

## Method

### Participants

A survey was conducted in February 2021 targeting faculty members from various universities in China. The recruitment period began on February 11, 2021, and concluded on February 25, 2021. Employing a convenience sampling approach, the questionnaire was distributed through wjx.cn, a popular online survey platform in China. Participants were informed about the purpose of the study, the voluntary nature of their participation, and the confidentiality of their responses.

### Written consent

Prior to completing the survey, participants were required to provide written consent. The consent process involved the following steps:

Firstly, participants were presented with an information sheet detailing the study’s objectives, procedures, potential risks and benefits, and the strict measures taken to ensure the confidentiality of their data. These measures included, but were not limited to, data encryption, secure storage, and restricted access to the collected information.

Secondly, written consent was obtained electronically through the survey platform, where participants had to actively check a box indicating their agreement to participate in the study, acknowledging that their data would be handled in accordance with the described confidentiality measures.

After rigorous data cleaning procedures, a total of 394 valid responses were included in the final analysis. The study was approved by the Ethics Committee of Hebei Finance University, School of Foreign Languages for International Business, and all procedures were in accordance with the ethical standards of the institution.

### Instruments

To assess Intrinsic Work Motivation (IWM), we adapted a scale grounded in Self-determination Theory from the Multidimensional Work Motivation Scale (MWMS) [64]. Three relevant items were selected for this study. For Value-Based Leadership (VBL), an updated version of the scale by [65] was utilized. Faculty’s Growth Mindset (GM) was measured using a 3-item scale developed by [19]. Teaching Self-Efficacy (TSE) was evaluated using a modified version of the scale by [66], based on Bandura’s Social Cognitive Theory. All measures employed a 7-point Likert scale ranging from 1 (strongly disagree) to 7 (strongly agree) (see S1 Questionnaire file for the complete questionnaire).

### Analysis

Partial Least Squares Structural Equation Modeling (PLS-SEM) was adopted as the primary analytical approach to examine both the measurement models and structural models. The measurement model portrays the relationships between latent variables and their manifest indicators, while the structural model represents the associations among the various constructs. Utilizing Structural Equation Modeling (SEM), a mediating analysis was conducted to explore the mediating role of faculty’s growth mindset and teaching self-efficacy. SmartPLS 3.0 software was used to conduct the analysis. To detect common method bias, Harman’s single factor test was implemented. By loading all items into a single common factor, the overall variance for this factor was found to be 39%, falling below the 50% threshold as suggested by [67]. This indicates that common method bias did not significantly impact the data or the study’s findings.

## Results

### Measurement model assessment

The reliability and validity of the constructs were rigorously evaluated to ensure the quality of the measurement model. The assessment encompassed several essential indicators, including internal consistency reliability, indicator reliability, convergent validity, and discriminant validity.

#### a. Internal consistency reliability

Cronbach’s alpha and composite reliability were calculated to assess the internal stability of the measures. As indicated in Table 1, all Cronbach’s alpha values exceeded the recommended threshold of 0.8, and composite reliability was greater than 0.9, demonstrating excellent internal consistency [68, 69]. These findings signify the high degree of reliability in the measurement scales.

**Table 1:** Reliability, indicator loadings and AVE (N = 394).

Construct/Item	Cronbach’s alpha	Composite reliability	AVE	Loading
**Intrinsic Work Motivation**	0.927	0.954	0.873	
IWM1. I make efforts getting involved in my job is because I have fun doing my job.				0.929
IWM2. I make efforts getting involved in my job is because what I do in my work is exciting.				0.948
IWM3. I make efforts getting involved in my job is because the work I do is interesting.				0.926
**Value-based Leadership**	0.984	0.985	0.774	
VBL1. My leader is truthful, honest, and displays moral behavior.				0.857
VBL2. My leader has healthy self-confidence and self-esteem.				0.882
VBL3. My leader does not lose sight of his or her goals or compromise on his or her principles.				0.841
VBL4. My leader has an inspiring vision.				0.847
VBL5. My leader finds ways to communicate his or her vision to his or her followers.				0.829
VBL6. My leader inspires trust and hope in his or her followers.				0.906
VBL7. My leader has the loyalty of the followers.				0.888
VBL8. My leader has a willingness to serve.				0.910
VBL9. My leader listens to his or her followers.				0.910
VBL10. My leader encourages dissenting opinion among his or her closest advisers.				0.885
VBL11. My leader is committed to the moral principle of respect for the followers.				0.910
VBL12. My leader includes the people affected in the change process.				0.923
VBL13. My leader is clear about his or her own beliefs e.g. assumptions about human nature, the role of the organization, the measurement of performance, etc.				0.929
VBL14. My leader listens to the needs, ideas, and aspirations of his or her followers and responds to them within the context of his or her well-developed systems of belief in the appropriate fashion.				0.926
VBL15. My leader has ideas.				0.715
VBL16. My leader shares information with his or her followers.				0.857
VBL17. My leader fosters a sense of community.				0.892
VBL18. My leader creates a consistent system of rewards, structure, process, and communication.				0.893
VBL19. My leader is committed to a principle of opportunity, giving all followers the chance to make a contribution to the organization.				0.887
**Teaching Self-efficacy**	0.915	0.931	0.627	
TSE1. I am convinced that I am able to successfully teach all relevant subject content to even the most difficult students.				0.799
TSE2. I know that I can maintain a positive relationship with parents even when tensions arise.				0.792
TSE3. When I try really hard, I am able to reach even the most difficult students.				0.782
TSE4. Even if I get disrupted while teaching, I am confident that I can maintain my composure and continue to teach well.				0.813
TSE5. I am confident in my ability to be responsive to my students’ needs even if I am having a bad day.				0.795
TSE6. If I try hard enough, I know that I can exert a positive influence on both the personal and academic development of my students.				0.816
TSE7. I know that I can motivate my students to participate in innovative projects.				0.795
TSE8. I know that I can carry out innovative projects even when I am opposed by skeptical colleagues.				0.740
**Growth Mindset**	0.856	0.912	0.776	
GM1. No matter who I am, I can change my intelligence a lot.				0.847
GM2. I can always greatly change how intelligent you are.				0.899
GM3. No matter how much intelligence I have, I can always change it quite a bit.				0.896

Source: the data is compiled based on the results obtained from SmartPLS 3.0 software.

#### b. Indicator reliability

The outer loadings of individual items onto their respective constructs were examined to evaluate indicator reliability. All items exhibited outer loadings greater than 0.7, and they were statistically significant at a 99% confidence level [69]. This confirms the reliability of the individual indicators in effectively capturing the latent constructs.

#### c. Convergent validity

To assess the extent to which measures of the same construct converge, the Average Variance Extracted (AVE) was computed for each construct. The AVE ranged from 0.627 to 0.873, exceeding the 0.5 threshold [69]. This indicates strong convergent validity, suggesting that the measures are capturing the intended constructs effectively.

#### d. Discriminant validity

To establish the distinctiveness of the constructs, discriminant validity was examined using two methods: cross-loadings and the Fornell-Larcker criterion. The cross-loadings analysis (Table 2) revealed that each item loaded higher on its intended construct than on any other construct, thereby confirming discriminant validity. Furthermore, the Fornell-Larcker criterion was met (Table 3), as the square root of each construct’s AVE exceeded its highest correlation with any other construct [69]. This provides further evidence of discriminant validity among the constructs.

**Table 2:** Discriminant validity by using crossing loadings (N = 394).

	Growth Mindset (GM)	Teaching Self-efficacy (TSE)	Value-based Leadership (VBL)	Intrinsic Work Motivation (IWM)
GM1	0.848	0.439	0.380	0.398
GM2	0.897	0.481	0.361	0.353
GM3	0.897	0.501	0.373	0.387
TSE1	0.454	0.799	0.372	0.418
TSE 2	0.407	0.792	0.394	0.432
TSE 3	0.359	0.782	0.362	0.445
TSE 4	0.424	0.813	0.365	0.412
TSE 5	0.460	0.795	0.402	0.366
TSE 6	0.400	0.816	0.429	0.468
TSE 7	0.397	0.795	0.358	0.430
TSE 8	0.505	0.740	0.401	0.372
VBL1	0.352	0.376	0.857	0.436
VBL2	0.371	0.406	0.882	0.469
VBL3	0.371	0.410	0.841	0.465
VBL4	0.431	0.456	0.847	0.451
VBL5	0.355	0.453	0.829	0.410
VBL6	0.390	0.441	0.906	0.420
VBL7	0.367	0.436	0.888	0.424
VBL8	0.353	0.424	0.910	0.426
VBL9	0.366	0.421	0.910	0.440
VBL10	0.357	0.418	0.885	0.423
VBL11	0.324	0.395	0.910	0.390
VBL12	0.377	0.441	0.923	0.467
VBL13	0.373	0.481	0.929	0.422
VBL14	0.398	0.473	0.926	0.422
VBL15	0.354	0.422	0.715	0.322
VBL16	0.360	0.375	0.857	0.380
VBL17	0.344	0.425	0.892	0.384
VBL18	0.384	0.440	0.893	0.431
VBL19	0.391	0.413	0.887	0.372
IWM1	0.307	0.447	0.420	0.925
IWM2	0.384	0.427	0.403	0.947
IWM3	0.345	0.474	0.344	0.931

Source: the data is compiled based on the results obtained from SmartPLS 3.0 software.

**Table 3:** Discriminant validity by using Fornell-Larcker criterion (N = 394).

	Growth Mindset	Teaching Self-efficacy	Value-based Leadership	Intrinsic Work Motivation
Growth Mindset	**0.881**			
Teaching Self-efficacy	0.537	**0.792**		
Value-based Leadership	0.421	0.488	**0.880**	
Intrinsic Work Motivation	0.370	0.482	0.415	**0.934**

Note: Square-root of AVE in bold; Source: the data is compiled based on the results obtained from SmartPLS 3.0 software.

Overall, the results of the reliability and validity tests conducted on the measurement model are deemed satisfactory, indicating that the items employed to measure the constructs in this study are valid and appropriate for use in the structural model.

### Structural model assessment

The structural model assessment serves as a critical step in validating the underlying theoretical framework by examining the empirical support for the proposed relationships among the constructs. The evaluation was conducted using key metrics such as path coefficients, coefficients of determination (R^2^), effect size (f^2^), and predictive relevance (Q^2^).

#### a. Path coefficients

**Fig 1:**
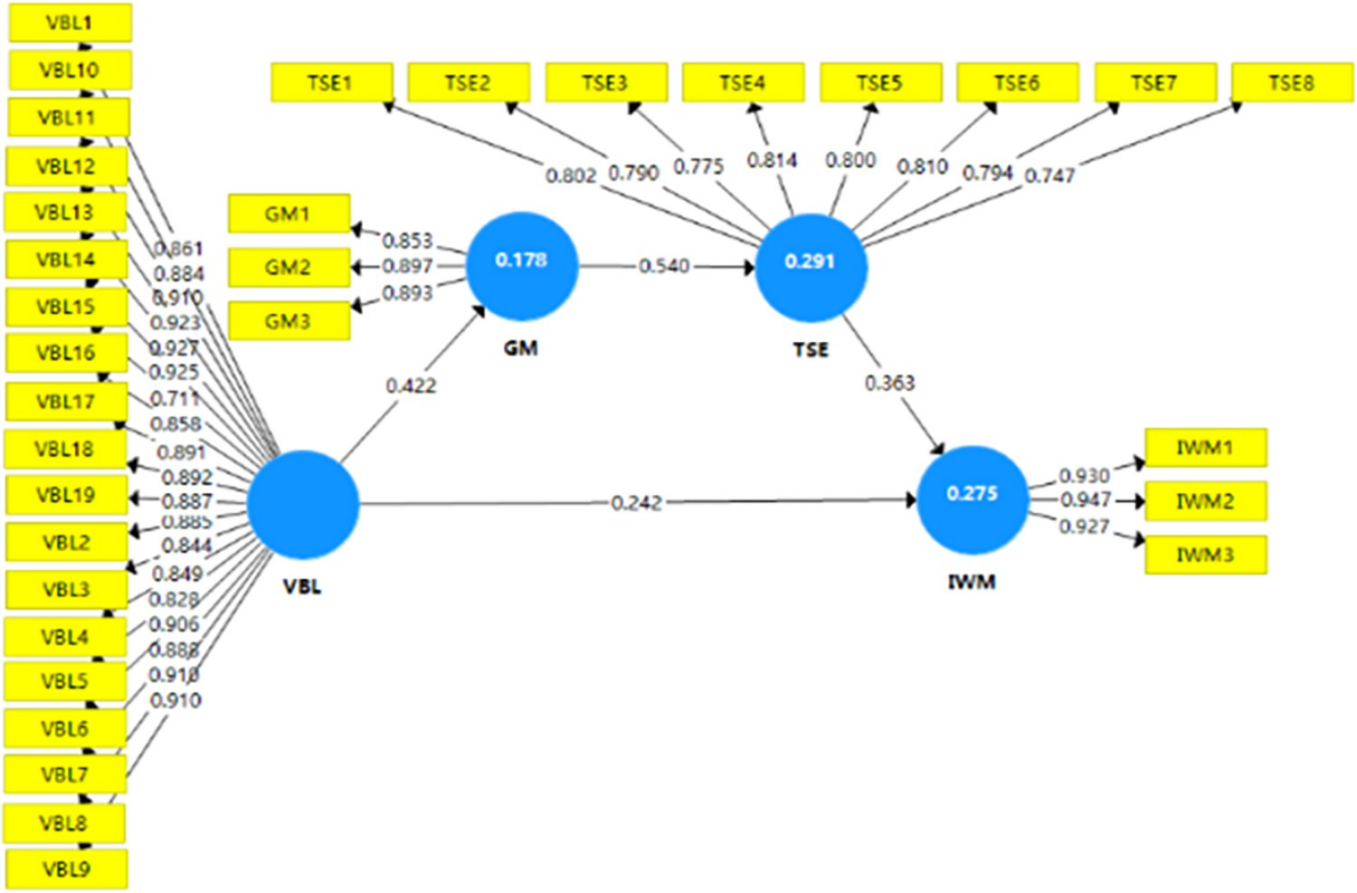
Path coefficients of the structural model. Source: the data is compiled based on the results obtained from SmartPLS 3.0 software.

The path coefficients were estimated to scrutinize the hypothesized relationships among the variables in the structural model. As presented in Fig 1 and Table 4, all relationships were found to be statistically significant at the 0.01 level, indicating robust associations among the constructs. The standardized path coefficients ranged from 0.242 to 0.540, demonstrating the strength and direction of the relationships within the model.

**Table 4:** Significance testing results of the structural model path coefficients.

	Path Coefficients	Sample Mean (M)	Standard Deviation (STDEV)	T Statistics (|O/STDEV|)	P Values	Significance Level
VBL → IWM	0.242	0.241	0.061	3.994	0.000	***
VBL→ GM	0.422	0.423	0.047	9.048	0.000	***
GM → TSE	0.540	0.540	0.042	12.938	0.000	***
TSE → IWM	0.363	0.364	0.062	5.851	0.000	***

Note

***p <0.01; Source: the data is compiled based on the results obtained from SmartPLS 3.0 software.

#### b. Coefficient of determination (R^2^)

The R^2^ values were computed to assess the predictive power of the exogenous variables on the endogenous variables. As shown in Table 5, the R^2^ values for Intrinsic Work Motivation (IWM), Teaching Self-efficacy (TSE), and Growth Mindset (GM) were 0.275, 0.291, and 0.178, respectively. These values signify moderate predictive power, with IWM and TSE being relatively better explained by the model compared to GM.

**Table 5:** Coefficients of determination of endogenous variables.

Endogenous Variables	R^2^ value	Criteria
IWM	0.275	Values of around 0.67, 0.33, and 0.19 are considered substantial, moderate, and weak predictive power respectively [70]
TSE	0.291	
GM	0.178	

Source: the data is compiled based on the results obtained from SmartPLS 3.0 software.

#### c. Effect size (f^2^)

The f^2^ effect sizes were calculated to quantify the substantiality of the impact of each exogenous variable on the endogenous variables. As reported in Table 6, all f^2^ values exceeded the low-effect threshold [71], with several exceeding the medium-effect threshold. These findings indicate that the exogenous variables in the model have substantial influence on the endogenous variables, supporting the validity of the structural model.

**Table 6:** Effect size of all exogenous variables.

Exogenous variables	f^2^ value	Criteria
VBL →IWM	0.062	f^2^ values of 0.02, 0.15, and 0.35, respectively suggest low, middle, and high level of effects [71]
VBL →GM	0.216	
GM → TSE	0.411	
TSE →IWM	0.138	

Source: the data is compiled based on the results obtained from SmartPLS 3.0 software.

#### d. Predictive relevance (Q^2^)

The Q^2^ values were used to ascertain the predictive relevance of the model for each endogenous construct. As evident from Table 7, the Q^2^ values for IWM, TSE, and GM were 0.236, 0.178, and 0.135, respectively, all exceeding zero. This validates the predictive significance of the model for each of the endogenous constructs, further corroborating the reliability and validity of the structural model.

**Table 7:** Predictive relevance of endogenous variables.

	SSO	SSE	Q^2^ (= 1-SSE/SSO)	Criteria
IWM	1182	903.222	0.236	Q^2^ >0 indicates the path model’s predictive relevance for this particular construct [69]
TSE	3152	2591.809	0.178	
GM	1182	1022.293	0.135	
VBL	7486	7486		

Source: the data is compiled based on the results obtained from SmartPLS 3.0 software.

#### e. Goodness-of-fit (GoF)

The GoF of an arithmetical model determines whether it fits well a set of observations. Values of GoF normally demonstrate the discrepancy between observed values and the predicted values in a particular model. According to [72], GoF can be calculated by the equation GoF = $\sqrt{\bar{AVE}}*\sqrt{\bar{R^{2}}}$, and the criteria of GoF is GoF_small_ = 0.1, GoF_medium_ = 0.25, and GoF_large_ = 0.36. In this study, based on the results of AVE (see Table 1) and R^2^ (see Table 5), GoF = $\sqrt{(0.873+0.774+0.627+0.776)/4}*\sqrt{(0.275+0.291+0.178)/3}$ ≈0.43, concluding that the model has a good performance in comparison to the baseline values specified above.

### Mediation analysis

To comprehend the intricacies of the proposed model, a mediation analysis was undertaken following the framework proposed by [73]. This analysis aimed to examine the indirect effects of Value-Based Leadership (VBL) on Intrinsic Work Motivation (IWM) through the serial mediation of Growth Mindset (GM) and Teaching Self-Efficacy (TSE).

Initially, the direct effect of VBL on IWM was assessed without considering the potential mediating variables. As depicted in Table 8, this direct relationship was found to be statistically significant (p < 0.01), suggesting the potential for mediation.

**Table 8:** Significance analysis of path coefficients.

	Path Coefficients	Sample Mean (M)	Standard Deviation (STDEV)	T Statistics (|O/STDEV|)	P Values	Significance Level
Excluding the mediator of TSE and GM						
VBL→IWM	0.422	0.424	0.049	8.550	0.000	***
Including the mediator of TSE and GM						
VBL → IWM	0.242	0.241	0.061	3.994	0.000	***
VBL→ GM	0.422	0.423	0.047	9.048	0.000	***
GM → TSE	0.540	0.540	0.042	12.938	0.000	***
TSE → IWM	0.363	0.364	0.062	5.851	0.000	***

Note: ***p <0.01; Source: the data is compiled based on the results obtained from SmartPLS 3.0 software.

Subsequently, the mediating variables (GM and TSE) were incorporated into the model to test the indirect effects. The prerequisite condition for mediation was established by verifying the significance of the paths between VBL and GM, GM and TSE, and TSE and IWM. As shown in the results, all these paths were statistically significant (p < 0.01).

The magnitude of the indirect effect was calculated as the product of the path coefficients along the serial mediation path (VBL → GM → TSE → IWM), yielding a value of 0.083 (0.422*0.540*0.363). This indirect effect was also found to be statistically significant (p < 0.01), as confirmed by bootstrapping analysis.

Furthermore, the Variance Accounted For (VAF) metric was utilized to assess the strength of the mediation. The direct effect of VBL on IWM was 0.242, while the indirect effect mediated by GM and TSE was 0.083. The total effect was calculated as the sum of these two effects (0.325). The VAF was then determined as the proportion of the indirect effect to the total effect, yielding a value of 0.255 (0.083/0.325). This indicates partial mediation, where 25.5% of the influence of VBL on IWM is explained through the serial mediation of GM and TSE.

Collectively, these findings confirm that the effect of VBL on IWM is partially mediated by the sequential influence of GM and TSE, elucidating the intricate mechanisms linking leadership style to faculty motivation.

## Discussion

The findings of this study offer a resounding response to the first research question, demonstrating a positive correlation between value-based leadership and faculty’s intrinsic work motivation. In simpler terms, superior value-based leadership anticipates heightened levels of faculty’s intrinsic work motivation. This discovery resonates with the findings of [52], who contend that values-based leadership fosters an atmosphere conducive to motivating group members, as employees perceive a sense of belonging and thrive in an encouraging work environment. Furthermore, [74] support this notion by elucidating the pivotal role of principals’ leadership in motivating teachers, suggesting that they can enhance teachers’ comprehension of their mission, function, and educational context by fostering an autonomous work environment.

This study also shows that value-based leadership fits well with the Chinese culture and society featured by high power-distance, relationship orientation, and collectivism. As noted by [18], high power-distance suggests that subordinates are more likely to be dependent heavily on their leaders and act in accordance with their leaders. Relationship-oriented society indicates a highlight on the interaction between leaders and subordinates. Collectivism prioritizes the common goal of group. Value-based leadership in Chinese cultural context is functional in facilitating faculty’s internalization of university goals into personal goals, feeling a solid ethical obligation to advance the performance of their organization by integrating the organizational values and goals into their educational activities [75].

The findings of this study answer the second research question by confirming that the effect of value-based leadership on faculty’s intrinsic work motivation is serially and partially mediated by faculty’s growth mindset and their teaching self-efficacy. Value-based leadership highlights building consensus with respect to the university’s vision, goals and priorities, allowing faculty a clear understanding of, and an agreement with, the university’s vision for the future [52], and thus they are more likely to have a growth mindset focusing on the process of pursuing goals, and develop their professional capabilities and skills, leading to an enhanced teaching self-efficacy.

The identification of growth mindset and teaching self-efficacy as serial mediators is able to explain how university leaders’ value-based leadership achieves its effect on faculty’s intrinsic work motivation, providing an idea about how universities can combine organizational factors such as leadership with individual factors such as growth mindset and teaching self-efficacy to enhance faculty’s intrinsic work motivation. It also allows a better comprehension of the correlations of value-based leadership and intrinsic work motivation via growth mindset and self-efficacy considering the complexity of motivation mechanism.

## Conclusion

Given the constraints imposed by the COVID-19 pandemic, this study resorted to convenience sampling for data collection. This method, while facilitating data gathering under challenging circumstances, introduced limitations regarding the generalizability of the findings [76]. Specifically, the use of convenience sampling may have restricted the representativeness of the results, impacting their external validity.

Additionally, the study’s exclusive focus on faculty’s intrinsic work motivation within the Chinese socio-cultural context, characterized by a bureaucratic managerial culture influenced by China’s governmental system [77], further limits the universal application of these results. The cultural specificities inherent in the observed leadership styles and motivational factors may not be directly transferable to other nations with divergent cultural landscapes.

To address these limitations, future research could take two primary directions. First, employing a more diverse or randomized sampling approach would strengthen the external validity of the findings, allowing for greater generalization across different populations. Second, investigating the cross-cultural applicability of the study’s results by exploring faculty motivation in other cultural contexts would provide valuable insights into the universality or cultural specificity of the observed phenomena. Despite the current constraints, the study’s insights still offer valuable theoretical and practical implications for researchers, university leaders, administrators, and policymakers, particularly within the Chinese socio-cultural context.

From a theoretical perspective, this research extends the existing literature on motivation by elucidating the substantial influence of value-based leadership on faculty’s intrinsic work motivation within the Chinese socio-cultural framework. It further enriches our understanding by proposing a sequential mediation model involving two mediators—growth mindset and teaching self-efficacy—that connects value-based leadership with faculty’s intrinsic motivation. Notably, few studies have delved into such an intricate model with dual serial mediators to uncover the underlying mechanisms linking value-based leadership and intrinsic work motivation.

Practically, this study contributes to a deeper comprehension of strategies to foster faculty’s intrinsic work motivation in higher education settings. The findings underscore the potential for university leaders to enhance faculty members’ intrinsic motivation by fostering value-based leadership. Specifically, leaders ought to cultivate supportive relationships among faculty members, fostering a collaborative environment characterized by unity, openness, and shared aspirations [53]. For instance, leaders could facilitate regular team-building activities such as workshops or retreats, encourage open communication channels through regular check-ins and feedback sessions, and establish shared goal-setting processes that involve faculty input and collaboration. By implementing these practices, faculty members are more likely to adopt a growth mindset and develop robust efficacy beliefs as they pursue common goals [63], ultimately propelling their intrinsic work motivation to new heights.

## Supporting information

S1 Questionnaire The questionnaire used to collect the data presented in this study. (DOCX)
