# Supplementary material for: Revolutionizing faculty’s intrinsic work motivation in China: A novel serial mediation model integrating value-based leadership, growth mindset, and teaching self-efficacy
Source: PLoS One. 2025 Jan 14;20(1):e0313392. doi: 10.1371/journal.pone.0313392 (PMC11731714; doi:10.1371/journal.pone.0313392)
Supplement: S1 Questionnaire — (DOCX) [file pone.0313392.s001.docx]

# Survey on Teachers’ Work Motivation in Chinese Universities

Dear participants:

This survey is part of a PhD candidate’s study of teachers’ work motivation in Chinese Universities and it attempts to provide helpful information for improving higher education in China. You are randomly selected and will not be identified by name. The survey will take approximately 10 - 15 minutes to complete. All information you provide will be treated as strictly confidential. Thank you for your contribution to this study.

**Part 1: Intrinsic Work Motivation**

I make efforts getting involved in my job is

|  | Strongly disagree | Disagree | Somewhat disagree | Neither agree nor disagree | Somewhat agree | Agree | Strongly agree |
| --- | --- | --- | --- | --- | --- | --- | --- |
| 1. Because I have fun doing my job. | 1 | 2 | 3 | 4 | 5 | 6 | 7 |
| 1. Because what I do in my work is exciting. | 1 | 2 | 3 | 4 | 5 | 6 | 7 |
| 1. Because the work I do is interesting. | 1 | 2 | 3 | 4 | 5 | 6 | 7 |

**Part 2: Value-based Leadership**

Your leader

|  | Strongly disagree | Disagree | Somewhat disagree | Neither agree nor disagree | Somewhat agree | Agree | Strongly agree |
| --- | --- | --- | --- | --- | --- | --- | --- |
| 1. Is truthful, honest, and displays moral behavior. | 1 | 2 | 3 | 4 | 5 | 6 | 7 |
| 1. Has healthy self-confidence and self-esteem. | 1 | 2 | 3 | 4 | 5 | 6 | 7 |
| 1. Does not lose sight of his or her goals or compromise on his or her principles. | 1 | 2 | 3 | 4 | 5 | 6 | 7 |
| 1. Has an inspiring vision. | 1 | 2 | 3 | 4 | 5 | 6 | 7 |
| 1. Finds ways to communicate his or her vision to his or her followers. | 1 | 2 | 3 | 4 | 5 | 6 | 7 |
| 1. Inspires trust and hope in his or her followers. | 1 | 2 | 3 | 4 | 5 | 6 | 7 |
| 1. Has the loyalty of the followers. | 1 | 2 | 3 | 4 | 5 | 6 | 7 |
| 1. Has a willingness to serve. | 1 | 2 | 3 | 4 | 5 | 6 | 7 |
| 1. Listens to his or her followers. | 1 | 2 | 3 | 4 | 5 | 6 | 7 |
| 1. Encourages dissenting opinion among his or her closest advisers. | 1 | 2 | 3 | 4 | 5 | 6 | 7 |
| 1. Is committed to the moral principle of respect for the followers. | 1 | 2 | 3 | 4 | 5 | 6 | 7 |
| 1. Includes the people affected in the change process. | 1 | 2 | 3 | 4 | 5 | 6 | 7 |
| 1. Is clear about his or her own beliefs e.g. assumptions about human nature, the role of the organization, the measurement of performance, etc. | 1 | 2 | 3 | 4 | 5 | 6 | 7 |
| 1. Listens to the needs, ideas, and aspirations of his or her followers and responds to them within the context of his or her well-developed systems of belief in the appropriate fashion. | 1 | 2 | 3 | 4 | 5 | 6 | 7 |
| 1. Has ideas. | 1 | 2 | 3 | 4 | 5 | 6 | 7 |
| 1. Shares information with his or her followers. | 1 | 2 | 3 | 4 | 5 | 6 | 7 |
| 1. Fosters a sense of community. | 1 | 2 | 3 | 4 | 5 | 6 | 7 |
| 1. Creates a consistent system of rewards, structure, process, and communication. | 1 | 2 | 3 | 4 | 5 | 6 | 7 |
| 1. Is committed to a principle of opportunity, giving all followers the chance to make a contribution to the organization. | 1 | 2 | 3 | 4 | 5 | 6 | 7 |

**Part 3: Teaching Self-efficacy**

|  | Strongly disagree | Disagree | Somewhat disagree | Neither agree nor disagree | Somewhat agree | Agree | Strongly agree |
| --- | --- | --- | --- | --- | --- | --- | --- |
| 1. I am convinced that I am able to successfully teach all relevant subject content to even the most difficult students. | 1 | 2 | 3 | 4 | 5 | 6 | 7 |
| 1. I know that I can maintain a positive relationship with parents even when tensions arise. | 1 | 2 | 3 | 4 | 5 | 6 | 7 |
| 1. When I try really hard, I am able to reach even the most difficult students. | 1 | 2 | 3 | 4 | 5 | 6 | 7 |
| 1. Even if I get disrupted while teaching, I am confident that I can maintain my composure and continue to teach well. | 1 | 2 | 3 | 4 | 5 | 6 | 7 |
| 1. I am confident in my ability to be responsive to my students’ needs even if I am having a bad day. | 1 | 2 | 3 | 4 | 5 | 6 | 7 |
| 1. If I try hard enough, I know that I can exert a positive influence on both the personal and academic development of my students. | 1 | 2 | 3 | 4 | 5 | 6 | 7 |
| 1. I know that I can motivate my students to participate in innovative projects. | 1 | 2 | 3 | 4 | 5 | 6 | 7 |
| 1. I know that I can carry out innovative projects even when I am opposed by skeptical colleagues. | 1 | 2 | 3 | 4 | 5 | 6 | 7 |

**Part 4: Growth Mindset**

|  | Strongly disagree | Disagree | Somewhat disagree | Neither agree nor disagree | Somewhat agree | Agree | Strongly agree |
| --- | --- | --- | --- | --- | --- | --- | --- |
| 1. No matter who you are, you can change your intelligence a lot. | 1 | 2 | 3 | 4 | 5 | 6 | 7 |
| 1. You can always greatly change how intelligent you are. | 1 | 2 | 3 | 4 | 5 | 6 | 7 |
| 1. No matter how much intelligence you have, you can always change it quite a bit. | 1 | 2 | 3 | 4 | 5 | 6 | 7 |

**Part 5: Demographic questions**

Please mark the answer with a “√” for EACH of the following questions.

1. You are: (a) a faculty member engaged in teaching exclusively

(b) an administrative staff without teaching engagement

(c) a faculty member engaged in both teaching and administration

1. Your gender: (a) Male (b) Female
2. Your age range: (a) Under 30 years old (b) 30-39 years old

(c) 40-49 years old (d) 50-59 years old

1. Your marital status: (a) Unmarried (b) Married without children (c) Married with children
2. The highest educational degree you’ve completed: (a) Bachelor (b) Master (c) PhD
3. Years of your teaching experience: (a) Under 3 years

(b) 3-10 years

(c) 11-20 years

(d) 21-30 years

(e) Over 30 years

40. Your academic rank: (a) Teaching assistant (b) Lecturer

(c) Associate Professor (d) Professor

41. Your academic discipline: (a) Formal science (mathematics, statistics, logic, etc.)

(b) Natural science (physics, chemistry, geology, biology, etc.)

(c) Applied science (engineering, medicine, etc.)

(d) Social science (economics, business, law, education, history, linguistics, etc.)

42. The college or university you work for is: (a) a public one titled with “985/211”

(b) a public one, neither “985” nor “211”

(c) a private one

43. Your personal disposal income: (a) less than ¥6,000/month

(b) ¥6,000 - ¥10,000/month

(c) more than ¥10,000/month
